# Efficacy of warm acupuncture combined with conventional therapy in patients with chronic glomerulonephritis

**DOI:** 10.1097/MD.0000000000047915

**Published:** 2026-03-13

**Authors:** Yatong Li, Zongjie Shang, Dingchao Wang, Shaochong Zhang

**Affiliations:** a Department of Intensive Care Unit, Traditional Chinese Medicine Hospital of Puyang City, Puyang City, Henan Province, China; b Department of Nephrology, Traditional Chinese Medicine Hospital of Puyang City, Puyang City, Henan Province, China.

**Keywords:** chronic glomerulonephritis, chronic kidney disease, estimated glomerular filtration rate, traditional Chinese medicine, warm acupuncture

## Abstract

This retrospective study evaluated the efficacy and safety of warm acupuncture combined with conventional therapy in patients with chronic glomerulonephritis. A total of 134 patients treated between May 2023 and May 2025 were included, with 69 receiving warm acupuncture plus conventional therapy (observation group) and 65 receiving conventional therapy alone (control group). Baseline demographic characteristics, comorbidities, renal function parameters, and proteinuria indices were comparable between groups. Renal function improved significantly after treatment in both groups; however, the observation group showed greater benefits, with a larger increase in estimated glomerular filtration rate (6.8 ± 5.5 vs 3.2 ± 5.0 mL/min/1.73 m^2^; *P* < .001) and more pronounced reductions in serum creatinine (−12.3 ± 10.0 vs −6.1 ± 9.5 μmol/L; *P* < .001) and blood urea nitrogen (−1.4 ± 1.1 vs −0.7 ± 1.0 mmol/L; *P* < .001). Proteinuria also decreased in both groups, with greater reductions in the observation group for 24-hour urinary protein (−0.96 ± 0.65 vs −0.61 ± 0.60 g/24 h; *P* = .001) and urinary protein-to-creatinine ratio (−0.82 ± 0.55 vs −0.48 ± 0.50 g/g; *P* < .001). The overall incidence of adverse events did not differ significantly between groups, and warm acupuncture-related local reactions were mild and self-limiting. These findings suggest that warm acupuncture is a safe and potentially useful adjunct to optimize renal protection and proteinuria control in chronic glomerulonephritis.

## 1. Introduction

Chronic glomerulonephritis (CGN) is a major cause of chronic kidney disease (CKD) and contributes substantially to the global burden of end-stage kidney disease and cardiovascular morbidity. Progressive loss of nephron mass in CGN is driven by persistent glomerular injury, hemodynamic maladaptation, and tubulointerstitial fibrosis, ultimately leading to irreversible decline in estimated glomerular filtration rate (eGFR). Recent epidemiological and cohort data have underscored that even moderate CKD confers a high risk of hospitalization, cardiovascular events, and mortality, emphasizing the need for early and effective renoprotective strategies.^[1,2]^ Proteinuria is a central marker and mediator of CGN progression. Elevated urinary protein excretion is strongly associated with accelerated eGFR decline and adverse renal and cardiovascular outcomes across CKD stages, and is therefore a key therapeutic target in contemporary management algorithms.^[3,4]^ Current guideline-based treatment focuses on blood pressure control, blockade of the renin–angiotensin–aldosterone system (RAAS), individualized immunosuppressive therapy according to glomerular disease subtype, and optimization of metabolic and lifestyle factors.^[5]^ Despite these measures, many patients maintain residual proteinuria and continue to experience progressive renal dysfunction, while long-term immunosuppression and multidrug regimens may increase the risk of adverse events (AEs) and treatment intolerance.^[6,7]^

In this context, integrative therapeutic approaches combining conventional medicine with evidence-based traditional Chinese medicine (TCM) have attracted increasing attention in nephrology. Several recent clinical and translational studies have suggested that specific Chinese herbal formulations can attenuate albuminuria, slow eGFR decline, and modulate inflammatory and fibrotic pathways in CKD, including glomerulonephritis.^[8,9]^ These findings support the concept that multi-target interventions may complement standard renoprotective strategies. However, most existing work has focused on oral herbal preparations, and high-quality data on external TCM modalities in CGN remain limited. Acupuncture-based interventions represent another important pillar of TCM and have been increasingly explored as adjuvant therapies in CKD. Recent clinical studies and systematic reviews indicate that acupuncture may improve residual renal function, reduce symptom burden, and enhance health-related quality of life in patients with CKD and dialysis-dependent kidney failure, with a generally favorable safety profile.^[10]^ In diabetic kidney disease, meta-analytic evidence suggests that acupuncture combined with conventional Western medicine can reduce proteinuria and improve renal function indices more effectively than pharmacotherapy alone, although heterogeneity in acupoint selection and trial quality remains a concern.^[11]^

Warm acupuncture, which integrates needling with localized thermal stimulation at selected acupoints, is hypothesized to enhance microcirculation, modulate neurohumoral regulation, and exert anti-inflammatory and antifibrotic effects. These mechanisms are theoretically relevant to the pathophysiology of CGN, where intrarenal hemodynamics, immune activation, and interstitial fibrosis are key drivers of progression. Nonetheless, clinical evidence specifically addressing the renoprotective effects and safety of warm acupuncture as an adjunct to conventional therapy in CGN is scarce. Against this background, the present study investigated whether adding warm acupuncture to standard nephrology care could further improve renal function and proteinuria control, while maintaining acceptable tolerability, in patients with CGN.

## 2. Methods

### 2.1. Study design

This retrospective study was approved by the Ethics Committee of Traditional Chinese Medicine Hospital of Puyang City (2025PYTCM0911). The requirement for written informed consent was waived by the Ethics Committee because the study used retrospective data collected during routine care and all data were analyzed in a de-identified manner. This retrospective study included consecutive patients with a confirmed diagnosis of CGN who received standardized medical treatment at the Department of Nephrology of our hospital between May 1, 2023 and May 1, 2025. According to the actual treatment regimen administered in clinical practice, patients were allocated to the observation group, which received warm acupuncture combined with conventional therapy, or to the control group, which received conventional therapy alone. Inclusion criteria were as follows: age between 18 and 75 years; diagnosis of CGN based on clinical manifestations, laboratory parameters, and/or renal biopsy findings consistent with current nephrology guidelines; disease course of at least 3 months before enrollment; baseline eGFR ≥ 30 mL/min/1.73 m^2^; and availability of complete clinical, laboratory, and follow-up data. Exclusion criteria included: acute exacerbation of glomerulonephritis, rapidly progressive glomerulonephritis, or end-stage renal disease; secondary glomerular diseases such as diabetic nephropathy, lupus nephritis, or vasculitis-associated nephritis; severe hepatic, cardiac, or hematologic dysfunction, active infection, or malignancy; pregnancy or lactation; receipt of acupuncture or moxibustion specifically for renal disease within 3 months prior to the index treatment; and incomplete medical records or loss to follow-up. Potential selection bias and confounding. Because treatment allocation was determined by real-world clinical decisions and patient preference rather than randomization, selection bias and residual confounding are possible. Patients who chose or were recommended warm acupuncture may differ from controls in unmeasured characteristics (e.g., health-seeking behavior, treatment adherence, symptom burden, socioeconomic status, or clinician perception of disease activity) that could affect renal outcomes. We therefore interpret between-group comparisons as associations rather than causal effects.

### 2.2. Treatment protocols

Patients in both groups received guideline-directed conventional therapy for CGN, and the observation group additionally underwent warm acupuncture. Conventional therapy was administered by attending nephrologists according to current nephrology practice and included optimization of blood pressure and proteinuria control with RAAS inhibitors (angiotensin-converting enzyme inhibitors or angiotensin II receptor blockers), diuretics as needed for edema, lipid-lowering agents, and, when indicated, immunosuppressive regimens such as corticosteroids and/or other immunosuppressants (e.g., cyclophosphamide, mycophenolate mofetil, or calcineurin inhibitors). Glycemic control was implemented in patients with concomitant diabetes mellitus, and all patients received general supportive measures including dietary counseling (salt and protein restriction as appropriate), smoking cessation advice, and weight management. Drug selection, dosage adjustment, and treatment duration were individualized based on renal function, blood pressure, comorbidities, and tolerance, but the overall therapeutic principles were consistent between the 2 groups.

In the observation group, warm acupuncture was provided as an adjunct to the above conventional therapy. All procedures were performed in the acupuncture clinic of the hospital by licensed TCM practitioners using sterile, single-use filiform needles. Commonly selected acupoints for tonifying kidney qi and promoting blood circulation included Shenshu (BL23), Guanyuan (CV4), Zusanli (ST36), Sanyinjiao (SP6), Taixi (KI3), and Mingmen (GV4), with minor individual adjustments permitted according to the patient’s constitution and symptom pattern. After routine skin disinfection, needles were inserted to an appropriate depth until the arrival of qi was elicited, followed by gentle manual manipulation. Warm stimulation was then applied by attaching moxa or a specialized warming device to the needle handles, maintaining a stable, tolerable local warmth without burning the skin. Each session lasted approximately 25 to 30 minutes, with needle retention throughout the warming period. In routine practice, warm acupuncture was performed 2 to 3 times per week over a continuous course of at least 4 weeks, and the total number of treatment sessions for each patient was recorded. Patients in the control group did not receive acupuncture, moxibustion, or any other TCM external therapy specifically targeting renal disease during the observation period.

### 2.3. Data collection

Clinical data were obtained retrospectively from the hospital electronic medical record system using a standardized data extraction form. For all eligible patients, baseline information was defined as the 1st visit at which the current treatment regimen (conventional therapy alone or conventional therapy combined with warm acupuncture) was initiated. Follow-up data were collected at the end of the predefined treatment course (post-treatment visit), which corresponded to the completion of warm acupuncture sessions in the observation group and the comparable time point in the control group. The following baseline demographic and clinical variables were recorded: age, sex, body mass index (BMI), disease duration of CGN, smoking and alcohol consumption history, systolic and diastolic blood pressure, and concomitant comorbidities (including hypertension, diabetes mellitus, and cardiovascular disease). Information on concomitant medications and therapeutic regimens was also collected, including use of RAAS inhibitors, diuretics, lipid-lowering agents, and immunosuppressive drugs, as well as detailed records of warm acupuncture prescriptions in the observation group.

Laboratory data were extracted at baseline and at the post-treatment visit. Renal function parameters included serum creatinine (Scr), blood urea nitrogen (BUN), and eGFR. eGFR was calculated using the Chronic Kidney Disease Epidemiology Collaboration (CKD-EPI) equation based on Scr, age, and sex. Proteinuria-related indices comprised 24-hour urinary protein excretion and the urinary protein-to-creatinine ratio (UPCR) measured in a spot urine sample. All biochemical measurements were performed in the hospital central laboratory using standardized automated assays and quality control procedures. Safety data were collected for the entire treatment period. All AEs, suspected drug-related reactions, and warm acupuncture-related local reactions were systematically recorded from inpatient and outpatient records. Serious adverse events (SAEs), reasons for treatment discontinuation or withdrawal, and outcomes of each event were documented. When necessary, missing or ambiguous entries were cross-checked by reviewing original charts or consulting the treating physicians to ensure data accuracy and completeness. The post-treatment visit reflected short-term follow-up after completion of the treatment course; therefore, long-term renal outcomes (e.g., CKD progression, end-stage renal disease, or long-term eGFR slope) could not be assessed.

### 2.4. Statistical analysis

All statistical analyses were performed using IBM SPSS Statistics, version 26.0 (IBM Corp., Armonk). Continuous variables (e.g., age, BMI, eGFR, Scr, BUN, 24-hour urinary protein, and urinary protein-to-creatinine ratio) were expressed as mean ± standard deviation, and categorical variables (e.g., sex, comorbidities, and AEs) were summarized as frequencies and percentages. Between-group comparisons of baseline continuous variables and of changes in renal function and proteinuria indices (ΔeGFR, ΔScr, ΔBUN, Δ24-hour urinary protein, and ΔUPCR) were conducted using independent-samples *t* tests. Within-group pre- to post-treatment comparisons of continuous outcomes were analyzed using paired-samples *t* tests. Categorical variables, including the prevalence of comorbidities and the incidence of overall AEs, specific drug-related adverse reactions, and warm acupuncture-related local reactions, were compared between groups using χ^2^ tests with continuity correction when appropriate. All statistical tests were 2-sided, and a *P* value < .05 was considered to indicate statistical significance.

## 3. Results

### 3.1. Baseline demographic and disease-related characteristics

A total of 134 patients with CGN were included in the analysis, with 69 allocated to the observation group and 65 to the control group. The mean age was 46.2 ± 11.3 years in the observation group and 45.7 ± 10.9 years in the control group (*t* = 0.26, *P* = .794), and the proportion of male patients was 55.1% (38/69) and 52.3% (34/65), respectively (*χ*^2^ = 0.10, *P* = .748). The mean disease duration was 5.3 ± 3.1 years in the observation group and 5.0 ± 3.0 years in the control group (*t* = 0.57, *P* = .569). Indices of general condition, including BMI, systolic blood pressure, and diastolic blood pressure, did not differ significantly between groups (all *P* > .05). The prevalence of major comorbidities was also similar. The proportions of patients with hypertension were 42.0% in the observation group and 40.0% in the control group (χ^2^ = 0.06, *P* = .811), while diabetes mellitus was present in 20.3% and 20.0% of patients, respectively (χ^2^ = 0.00, *P* = .967). Cardiovascular disease was documented in 15.9% of patients in the observation group and 13.8% in the control group (χ^2^ = 0.12, *P* = .734). Baseline renal function parameters were balanced between groups. The mean eGFR was 66.4 ± 14.2 mL/min/1.73 m^2^ in the observation group and 65.7 ± 13.8 mL/min/1.73 m^2^ in the control group (*t* = 0.29, *P* = .772). Scr (94.6 ± 18.5 vs 95.2 ± 19.1 μmol/L; *t* = −0.19, *P* = .854) and BUN (7.1 ± 1.8 vs 7.3 ± 1.9 mmol/L; *t* = −0.63, *P* = .532) were likewise comparable. The baseline 24-hour urinary protein excretion was 2.58 ± 0.96 g/24 h in the observation group and 2.63 ± 0.92 g/24 h in the control group (*t* = −0.31, *P* = .758) (Table 1).

**Table 1:** Baseline demographic and clinical characteristics of the study population.

Variable	Observation group (n = 69)	Control group (n = 65)	*t*/χ^2^	*P* value
Age, mean ± SD (yr)	46.2 ± 11.3	45.7 ± 10.9	*t* = 0.26	.794
Male sex, n (%)	38 (55.1)	34 (52.3)	χ^2^ = 0.10	.748
Disease duration, years, mean ± SD	5.3 ± 3.1	5.0 ± 3.0	*t* = 0.57	.569
BMI, mean ± SD (kg/m^2^)	23.7 ± 2.8	23.5 ± 2.9	*t* = 0.41	.685
SBP, mean ± SD (mm Hg)	129.6 ± 12.4	130.8 ± 11.9	*t* = −0.57	.568
DBP, mean ± SD (mm Hg)	79.8 ± 8.3	80.2 ± 8.1	*t* = −0.28	.778
Hypertension, n (%)	29 (42.0)	26 (40.0)	χ^2^ = 0.06	.811
Diabetes mellitus, n (%)	14 (20.3)	13 (20.0)	χ^2^ = 0.00	.967
Cardiovascular disease, n (%)	11 (15.9)	9 (13.8)	χ^2^ = 0.12	.734
eGFR, mean ± SD (mL/min/1.73 m^2^)	66.4 ± 14.2	65.7 ± 13.8	*t* = 0.29	.772
Serum creatinine, mean ± SD (μmol/L)	94.6 ± 18.5	95.2 ± 19.1	*t* = −0.19	.854
Blood urea nitrogen, mean ± SD (mmol/L)	7.1 ± 1.8	7.3 ± 1.9	*t* = −0.63	.532
24-h urinary protein, mean ± SD (g/24 h)	2.58 ± 0.96	2.63 ± 0.92	*t* = −0.31	.758

BMI = body mass index, BUN = blood urea nitrogen, DBP = diastolic blood pressure, eGFR = estimated glomerular filtration rate, SBP = systolic blood pressure, SD = standard deviation.

### 3.2. Changes in renal function parameters between groups

The mean eGFR was 66.4 ± 14.2 mL/min/1.73 m^2^ in the observation group and 65.7 ± 13.8 mL/min/1.73 m^2^ in the control group (*P* = .773). Scr (94.6 ± 18.5 vs 95.2 ± 19.1 μmol/L; *P* = .854) and BUN (7.1 ± 1.8 vs 7.3 ± 1.9 mmol/L; *P* = .533) also showed no statistically significant differences between groups at baseline. Within-group comparisons showed that renal function improved significantly after treatment in both groups. In the observation group, eGFR increased from 66.4 ± 14.2 to 73.2 ± 13.9 mL/min/1.73 m^2^, corresponding to a mean change (ΔeGFR) of 6.8 ± 5.5 mL/min/1.73 m^2^ (*t* = 10.27, *P* < .001). Scr decreased from 94.6 ± 18.5 to 82.3 ± 17.2 μmol/L, with a mean change (ΔScr) of −12.3 ± 10.0 μmol/L (*t* = −10.22, *P* < .001). BUN declined from 7.1 ± 1.8 to 5.7 ± 1.6 mmol/L, yielding a mean change (ΔBUN) of −1.4 ± 1.1 mmol/L (*t* = −10.57, *P* < .001). In the control group, eGFR increased from 65.7 ± 13.8 to 68.9 ± 13.5 mL/min/1.73 m^2^, with a mean ΔeGFR of 3.2 ± 5.0 mL/min/1.73 m^2^ (*t* = 5.16, *P* < .001). Scr decreased from 95.2 ± 19.1 to 89.1 ± 18.0 μmol/L, corresponding to a ΔScr of −6.1 ± 9.5 μmol/L (*t* = −5.18, *P* < .001). BUN decreased from 7.3 ± 1.9 to 6.6 ± 1.8 mmol/L, with a ΔBUN of −0.7 ± 1.0 mmol/L (*t* = −5.64, *P* < .001). These findings indicate that conventional therapy alone was associated with a statistically significant improvement in renal function. When comparing the magnitude of improvement between groups, the observation group showed a more pronounced enhancement in renal function. The mean increase in eGFR was greater in the observation group than in the control group (6.8 ± 5.5 vs 3.2 ± 5.0 mL/min/1.73 m^2^; *t* = 3.97, *P* < .001). The reduction in Scr was also more marked in the observation group (−12.3 ± 10.0 vs −6.1 ± 9.5 μmol/L; *t* = −3.68, *P* < .001). Similarly, the decrease in BUN was significantly larger in the observation group compared with the control group (−1.4 ± 1.1 vs −0.7 ± 1.0 mmol/L; *t* = −3.86, *P* < .001) (Table 2).

**Table 2:** Changes in renal function parameters before and after treatment in the 2 groups.

Variable	Time point	Observation group (n = 69) mean ± SD	Control group (n = 65) mean ± SD	*P* value
eGFR (mL/min/1.73 m^2^)	Baseline	66.4 ± 14.2	65.7 ± 13.8	.773
	Post-treatment	73.2 ± 13.9	68.9 ± 13.5	.072
	ΔeGFR (post − baseline)	6.8 ± 5.5	3.2 ± 5.0	<.001
Serum creatinine (μmol/L)	Baseline	94.6 ± 18.5	95.2 ± 19.1	.854
	Post-treatment	82.3 ± 17.2	89.1 ± 18.0	.027
	ΔScr (post − baseline)	−12.3 ± 10.0	−6.1 ± 9.5	<.001
Blood urea nitrogen (mmol/L)	Baseline	7.1 ± 1.8	7.3 ± 1.9	.533
	Post-treatment	5.7 ± 1.6	6.6 ± 1.8	.003
	ΔBUN (post − baseline)	−1.4 ± 1.1	−0.7 ± 1.0	<.001

eGFR = estimated glomerular filtration rate, SD = standard deviation.

### 3.3. Proteinuria and related urinary parameter changes

The mean 24-hour urinary protein excretion was 2.58 ± 0.96 g/24 h in the observation group and 2.63 ± 0.92 g/24 h in the control group (*P* = .759). Baseline UPCR values were 2.10 ± 0.75 g/g creatinine and 2.15 ± 0.78 g/g creatinine, respectively (*P* = .706), indicating no significant differences in proteinuria burden before treatment. Within-group analyses showed a significant reduction in proteinuria after treatment in both groups. In the observation group, 24-hour urinary protein decreased from 2.58 ± 0.96 to 1.62 ± 0.80 g/24 h, corresponding to a mean change (Δ24-hour urinary protein) of −0.96 ± 0.65 g/24 h (*t* = −12.27, *P* < .001). In the control group, 24-hour urinary protein decreased from 2.63 ± 0.92 to 2.02 ± 0.88 g/24 h, with a mean change of −0.61 ± 0.60 g/24 h (*t* = −8.20, *P* < .001). A similar pattern was observed for UPCR. In the observation group, UPCR decreased from 2.10 ± 0.75 to 1.28 ± 0.60 g/g creatinine, yielding a mean change (ΔUPCR) of −0.82 ± 0.55 g/g creatinine (*t* = −12.38, *P* < .001). In the control group, UPCR decreased from 2.15 ± 0.78 to 1.67 ± 0.65 g/g creatinine, with a mean ΔUPCR of −0.48 ± 0.50 g/g creatinine (*t* = −7.74, *P* < .001). These findings indicate that conventional therapy alone led to a statistically significant, albeit more modest, improvement in proteinuria. Between-group comparisons of the magnitude of change demonstrated that the observation group experienced a more pronounced reduction in proteinuria than the control group. The decrease in 24-hour urinary protein was significantly greater in the observation group than in the control group (−0.96 ± 0.65 vs −0.61 ± 0.60 g/24 h; *t* = −3.24, *P* = .001). Likewise, the reduction in UPCR was more marked in the observation group (−0.82 ± 0.55 vs −0.48 ± 0.50 g/g creatinine; *t* = −3.75, *P* < .001) (Table 3).

**Table 3:** Changes in proteinuria and related urinary parameters before and after treatment in the 2 groups.

Variable	Time point	Observation group (n = 69) mean ± SD	Control group (n = 65) mean ± SD	*P* value
24-h urinary protein (g/24 h)	Baseline	2.58 ± 0.96	2.63 ± 0.92	.759
	Post-treatment	1.62 ± 0.80	2.02 ± 0.88	.007
	Δ24-h urinary protein (g/24 h) (post − baseline)	−0.96 ± 0.65	−0.61 ± 0.60	.001
Urinary protein-to-creatinine ratio (UPCR, g/g creatinine)	Baseline	2.10 ± 0.75	2.15 ± 0.78	.706
	Post-treatment	1.28 ± 0.60	1.67 ± 0.65	<.001
	ΔUPCR (g/g creatinine) (post − baseline)	−0.82 ± 0.55	−0.48 ± 0.50	<.001

SD = standard deviation, UPCR = urinary protein-to-creatinine ratio.

### 3.4. Safety evaluation and AEs

The overall incidence of at least 1 AE was 31.9% (22/69) in the observation group and 24.6% (16/65) in the control group, and the difference was not statistically significant (χ^2^ = 0.55, *P* = .459). Drug-related adverse reactions were comparable between groups. The incidence of hypotension was 7.2% (5/69) in the observation group and 9.2% (6/65) in the control group (χ^2^ = 0.01, *P* = .918). Electrolyte disturbances occurred in 5.8% (4/69) and 7.7% (5/65) of patients, respectively (χ^2^ = 0.01, *P* = .926). Gastrointestinal reactions, including nausea, vomiting, and abdominal discomfort, were reported in 11.6% (8/69) of patients in the observation group and 10.8% (7/65) in the control group (χ^2^ = 0.00, *P* = 1.000). All drug-related AEs were mild to moderate in intensity and were controlled by symptomatic management or adjustment of concomitant medications without the need for treatment discontinuation. Warm acupuncture-related local reactions were observed only in the observation group and were predominantly mild. Local erythema at the needle insertion sites occurred in 5.8% (4/69) of patients, and transient local pruritus in 4.3% (3/69); 1 patient (1.4%) experienced a small superficial burn. These local reactions resolved spontaneously or with simple topical care and did not necessitate interruption or cessation of warm acupuncture. The differences in the incidence of local erythema (χ^2^ = 2.14, *P* = .143), pruritus (χ^2^ = 1.25, *P* = .264), and superficial burn (χ^2^ = 0.00, *P* = 1.000) between the 2 groups were not statistically significant. No deep burns, local infections, or other serious complications attributable to warm acupuncture were observed. SAEs were rare. One patient (1.4%) in the observation group and 2 patients (3.1%) in the control group experienced SAEs (such as acute cardiovascular or infectious complications) related to underlying comorbid conditions rather than to the study interventions; the difference between groups was not significant (χ^2^ = 0.00, *P* = .958). No deaths occurred during the observation period. Withdrawal due to AEs was uncommon, with no withdrawals in the observation group and 1 withdrawal (1.5%) in the control group (χ^2^ = 0.00, *P* = .976) (Table 4).

**Table 4:** Adverse events and safety outcomes in the 2 groups.

Adverse event	Observation group (n = 69) n (%)	Control group (n = 65) n (%)	χ^2^	*P* value
Any AE	22 (31.9)	16 (24.6)	0.55	.459
Drug-related adverse reactions				
Hypotension	5 (7.2)	6 (9.2)	0.01	.918
Electrolyte disturbance	4 (5.8)	5 (7.7)	0.01	.926
Gastrointestinal reactions (nausea, vomiting, abdominal discomfort)	8 (11.6)	7 (10.8)	0.00	1.000
Warm acupuncture-related local reactions				
Local erythema at needle site	4 (5.8)	0 (0.0)	2.14	.143
Local pruritus	3 (4.3)	0 (0.0)	1.25	.264
Superficial burn	1 (1.4)	0 (0.0)	0.00	1.000
SAEs	1 (1.4)	2 (3.1)	0.00	.958
Withdrawal due to AE	0 (0.0)	1 (1.5)	0.00	.976

AE = adverse event, SAE = serious adverse event.

## 4. Discussion

The present study evaluated the efficacy and safety of warm acupuncture combined with conventional therapy in patients with CGN. Both treatment regimens were associated with statistically significant improvements in renal function and proteinuria; however, the magnitude of improvement was consistently greater in the group receiving adjunctive warm acupuncture. At similar baseline levels of renal impairment, patients in the observation group exhibited larger increases in eGFR and more pronounced reductions in Scr and BUN compared with those receiving conventional therapy alone. In parallel, the decreases in 24-hour urinary protein excretion and UPCR were also significantly larger in the observation group, indicating superior control of glomerular protein leakage. Importantly, these benefits were achieved without an increase in systemic adverse drug reactions or serious complications, and warm acupuncture-related local reactions were mild and self-limiting.

From a pathophysiological perspective, the observed renal benefits are biologically plausible. CGN is characterized by persistent immune-mediated glomerular injury, intrarenal hemodynamic disturbance, and progressive interstitial fibrosis. Conventional therapy aims to control blood pressure and proteinuria, modulate immune activity, and optimize metabolic and cardiovascular risk factors. Warm acupuncture, as an external TCM modality, may exert complementary effects through several mechanisms. Experimental and clinical data suggest that acupuncture can modulate autonomic balance, improve renal microcirculation, and attenuate inflammatory and oxidative stress pathways, thereby slowing structural damage and preserving nephron function.^[12,13]^ Warm stimulation at kidney- and spleen-related acupoints may additionally enhance local perfusion, regulate neurohumoral responses, and influence RAAS activity, which could contribute to both improved filtration capacity (reflected by increased eGFR) and reduced glomerular permeability (reflected by lower proteinuria).

The greater reduction in proteinuria observed in the warm acupuncture group is particularly relevant, as proteinuria is a key surrogate marker and driver of CKD progression. Persistent proteinuria promotes tubular injury, inflammatory activation, and interstitial fibrosis. The combination of standard renoprotective drugs with non-pharmacological interventions that further reduce protein excretion may therefore have cumulative benefits. In this study, the adjunctive warm acupuncture group achieved nearly 1 g/24 h reduction in 24-hour urinary protein, significantly exceeding the reduction seen with conventional therapy alone. This indicates that warm acupuncture may act as a useful adjunct to optimize proteinuria control when pharmacological measures alone are insufficient or poorly tolerated. The safety profile observed in this cohort is also notable. The incidence and pattern of systemic adverse drug reactions, including hypotension, electrolyte disturbances, and gastrointestinal symptoms, were similar in the 2 groups, suggesting that warm acupuncture did not increase the burden of drug-related toxicity. Local reactions such as erythema, pruritus, and superficial burns were infrequent, mild, and reversible, and no deep burns, local infections, or treatment-related SAEs were recorded. These findings align with existing evidence indicating that acupuncture is generally safe when performed by trained practitioners and that the incidence of serious complications is very low.^[14]^

Although data specifically targeting warm acupuncture in CGN remain limited, the present results are consistent with and extend recent evidence on acupuncture-based interventions in CKD and related conditions. A randomized controlled trial protocol by Gao et al proposed that warm acupuncture combined with standard Western medicine could improve renal outcomes in type 2 diabetic kidney disease by reducing albuminuria and modulating key nephroprotective pathways; preliminary reports from this program suggest favorable trends in renal function and safety.^[15]^ A recent strategic analysis of acupuncture for diabetic kidney disease similarly concluded that acupuncture combined with conventional medications may reduce proteinuria and improve metabolic control, although heterogeneity in acupoint selection and treatment schemes was noted.^[11]^ Several systematic reviews and meta-analyses published in the past 3 years have provided quantitative support for the renoprotective role of acupuncture in CKD. Liu et al conducted a comprehensive meta-analysis and reported that acupuncture therapy significantly reduced Scr levels and improved certain kidney-related symptoms in patients with CKD, with an acceptable safety profile, although the overall certainty of evidence was rated as low to very low due to methodological limitations of the included trials. Wu et al analyzed randomized and quasi-randomized trials in diabetic nephropathy and found that acupuncture, particularly when used as an adjunct to Western medicine, was associated with reductions in proteinuria and improvements in renal function indices compared with standard care alone.^[16,17]^ These findings are congruent with the present observation that adding warm acupuncture to conventional therapy yields additional benefits in both eGFR and proteinuria.

From a broader TCM perspective, recent reviews of TCM-based protocols for CKD have emphasized that multi-target interventions, including herbal prescriptions, moxibustion, and acupuncture, can reduce proteinuria, mitigate inflammatory and fibrotic injury, and improve quality of life.^[18,19]^ A systematic review of moxibustion as an adjuvant therapy in CKD suggested that it may reduce Scr, BUN, and urinary protein excretion, though effects on eGFR were less consistent and the quality of evidence was limited.^[20]^ The current study, using warm acupuncture (which integrates acupuncture needling with moxibustion-like thermal stimulation), observed simultaneous improvements in both filtration indices and proteinuria, suggesting that combined mechanical and thermal stimulation at specific kidney-related acupoints may confer more comprehensive renal benefits than moxibustion alone. Furthermore, mechanistic reviews published in the last 3 years have summarized how acupuncture may influence key CKD-related pathways, including modulation of inflammatory cytokines, reduction of oxidative stress, improvement of endothelial function, and regulation of autonomic and hypothalamic–pituitary–adrenal axis activity. The pattern of enhanced eGFR, reduced nitrogenous waste, and attenuated proteinuria observed in the present study is compatible with these mechanistic insights.^[21,22]^ However, because the present study did not include biomarker or histopathological analyses, the exact pathways through which warm acupuncture exerted its effects in CGN remain to be clarified in future trials.

However, several limitations should be acknowledged. First, this was a retrospective, single-center study with non-randomized treatment allocation, which may introduce selection bias and residual confounding. The direction of bias is uncertain. Second, the warm acupuncture regimen allowed minor individualized acupoint adjustments based on constitution and symptom patterns. While this reflects real-world TCM practice, it increases intervention heterogeneity and may reduce reproducibility, making it difficult to determine whether outcomes were driven by the core point set, specific auxiliary points, or the overall treatment context. Future studies should adopt a more standardized protocol and report detailed treatment parameters (acupoints, stimulation, session frequency, total sessions) to improve comparability. On one hand, patients electing warm acupuncture may be more health-conscious and adherent to medications and lifestyle advice, which could inflate the apparent benefit of warm acupuncture. On the other hand, warm acupuncture may be preferentially offered to patients with greater symptom burden or poorer perceived control, which could attenuate the observed effect. Although baseline demographic and laboratory indices were comparable between groups, unmeasured factors (e.g., histopathologic subtype, inflammatory activity, socioeconomic status, and adherence) could still influence outcomes. Future multicenter prospective studies, ideally randomized controlled trials or well-designed observational studies using propensity-score methods, are needed to better control confounding and clarify causality. Accordingly, our results should be interpreted as short-term improvement trends in renal function and proteinuria rather than evidence of long-term renoprotection.

## 5. Conclusion

In conclusion, warm acupuncture combined with conventional therapy significantly improved renal function and reduced proteinuria in patients with CGN compared with conventional therapy alone, while maintaining a comparable systemic safety profile. Local warm acupuncture-related reactions were mild and self-limiting. These findings suggest that warm acupuncture is a feasible and safe adjunctive strategy to optimize renal protection in this population.

## Author contributions

**Conceptualization:** Yatong Li, Zongjie Shang, Dingchao Wang, Shaochong Zhang.

**Data curation:** Yatong Li, Zongjie Shang, Dingchao Wang, Shaochong Zhang.

**Formal analysis:** Yatong Li, Zongjie Shang, Dingchao Wang, Shaochong Zhang.

**Funding acquisition:** Yatong Li, Dingchao Wang, Shaochong Zhang.

**Investigation:** Shaochong Zhang.

**Writing – original draft:** Shaochong Zhang.

**Writing – review & editing:** Shaochong Zhang.
